# *Linc0023*9 Facilitates the Progress of Clear Cell Renal Cell Carcinoma via the miR-204-5p/*RAB22A* Axis

**DOI:** 10.1007/s12033-024-01202-w

**Published:** 2024-06-08

**Authors:** Cheng Cheng, Shuangquan Lin, Anyi Zhu, Zhengdong Hong, Zimin Shi, Huanhuan Deng, Gan Zhang

**Affiliations:** Department of Urology, The Second Affiliated Hospital of Nanchang University, No. 1 Minde Road, Nanchang, 330006 Jiangxi China

**Keywords:** Clear cell renal cell carcinoma, *Linc00239*, miR-204-5p, *RAB22A*, Progression

## Abstract

Long intergenic non-coding RNA 239 (*Linc00239*) acts as an oncogene in colorectal cancer (CRC), esophageal squamous cell carcinoma, and acute myeloid leukemia cells. However, its role and regulatory mechanisms in clear cell renal cell carcinoma (ccRCC) remain unknown. We used StarBase and The Cancer Genome Atlas databases to evaluate *Linc00239* expression and its effect on ccRCC. Furthermore, the function of *Linc00239* in ccRCC proliferation and metastasis was analyzed using Cell Counting Kit-8 and Transwell assays following *Linc00239* knockdown. Subsequently, the *Linc00239*-miRNA-mRNA regulatory associations were selected based on miRanda, miTarbase, and previous references, and their expression levels and binding relationship were further validated using quantitative real-time polymerase chain reaction, western blotting and dual-luciferase reporter gene assay. Additionally, we transfected a miRNA inhibitor to evaluate whether the miR-204-5p/*RAB22A* (Ras-related proteins in brain 22a) axis was involved in *Linc00239* function. *Linc00239* was elevated in ccRCC and correlated with poor prognosis. *Linc00239* knockdown inhibited ccRCC progression. Additionally, *Linc00239* inhibition elevated miR-204-5p expression and repressed *RAB22A* levels. Moreover, miR-204-5p inhibitors attenuated this inhibitory effect on proliferation, migration, invasion, and *RAB22A* level when *Linc00239* was knocked down. *Linc00239* promotes ccRCC proliferation and metastasis by elevating *RAB22A* expression through the adsorption of miR-204-5p, which provides a clue for the diagnosis and treatment of ccRCC.

## Introduction

Renal cell carcinoma is a type of malignancy of the urinary system, with 80%–90% of its cases diagnosed as clear cell renal cell carcinoma (ccRCC) according to pathological patterns [1, 2]. Current treatment strategies include radiotherapy and chemotherapy, with nearly one-third of patients being treated surgically with localized or distant metastasis at the time of initial diagnosis [1]. Although the survival rate has improved, most patients eventually succumb to this disease [3].

Long noncoding RNAs (lncRNAs) are a type of RNA with transcripts longer than 200 nt [1]. Previous studies have reported that lncRNAs play an important role in the progress of various cancers by affecting cell proliferation, apoptosis, and metastasis [4]. LncRNAs play a functional role by regulating the expression of microRNA (miRNA) and their target messenger RNA (mRNA), which is referred to as the competing endogenous RNA (ceRNA) mechanism [5]. For example, the lncRNA XIST suppresses the proliferation, invasion, migration, and colony formation of ovarian cancer cells but promotes their apoptosis by regulating the expressions of miR-149-3p and its target mRNA forkhead box P3 [6]. Betulinic acid inhibits lncRNA *MALAT1* expression to suppress the apoptosis of hepatocellular cancer cells via miR-22-3p and inhibitor of apoptosis proteins (IAPs) [7]. The lncRNA *MALAT1* serves as a sponge to regulate CRC cell function, such as survival, migration, and epithelial-mesenchymal transition (EMT) [8]. Additionally, studies have reported the involvement of lncRNAs in ccRCC progression. lncRNA *DNAJC3*-AS1 facilitates ccRCC progress by regulating miR-27a-3p and its target gene PR domain-containing protein 14 [9]. LINC01232 promotes ccRCC progression by sponging miR-204-5p and upregulating RAB22A [10]. Moreover, IncTCL6 impairs the ability of ccRCC to proliferate and migrate, thereby regulating the Src-AKT-mediated EMT pathway [11]. LncRNAs, such as lncRNA *ZNF180*-2 and lnc-*OTUD6B*-AS1 [1, 3], have been reported as prognostic biomarkers for ccRCC.

*Linc00239* was previously demonstrated as an oncogenic lncRNA in CRC [12–14], esophageal squamous cell carcinoma [15], and acute myeloid leukemia cells [16]. However, to the best of our knowledge, there have been no reports regarding its role and detailed regulatory mechanisms in ccRCC. Accordingly, this study explores the role and potential mechanism of Linc00239 to further aid the diagnosis and treatment of ccRCC.

## Methods

### Database

To determine the *Linc00239* expression and its relationship with selected mircoRNAs in ccRCC, we selected StarBase (http://StarBase.sysu.edu.cn/panGeneDiffExp.php) for analysis. The overall and disease-free survivals of *Linc00239* were analyzed using The Cancer Genome Atlas (TCGA) (http://gepia.cancer-pku.cn/detail.php?gene=Linc00239###). For the *Linc00239*–microRNA–mRNA network, microRNA was predicted using miRanda and miTarbase, among which four microRNAs were selected according to the reference reported in ccRCC. Then, the target mRNA of four microRNAs was predicted using miRanda and miTarbase. Finally, the *Linc00239*–microRNA–mRNA network was constructed using Cytoscape.

### Cell culture and transfection

HEK-293, 769-P, Caki-2, ACHN, SN12C, and 786-O were purchased from Cellcook (Guangzhou, China). Among them, HEK-293 and SN12C were cultured in DMEM (Gibco, Grand Island, NY, USA), Caki-2 in McCoy’s media (Gibco), ACHN in MEM, and 769-P and 786-O in RPMI 1640 (Gibco); all the media contained 10% fetal bovine serum (FBS) (Atlantic Biologicals, Atlanta, GA). To facilitate this process, a 5% CO_2_ humidified atmosphere at 37 °C was utilized to expand all the cells.

For the transfection of SN12C and 786-O, miR-204-5p inhibitor, siRNAs of *Linc00239* (si-*Linc00239*), and si-NC were procured from Genepharma (Shanghai, China). An overnight seeding of SN12C and 786-O cell suspension was conducted. Cell transfection was performed when cells were 70%–90% confluent. Then, two tubes were prepared; one was used to prepare a diluted lipofectamine 3000 reagent in MEM media, and the other was used to prepare dilute siRNAs or inhibitors. Subsequently, the dilute siRNAs or inhibitors were added to the dilute lipofectamine 3000 reagent (1:1) for 5 min and mixed. The cells were collected for further analysis after 48 h of incubation. The transfection was conducted using Lipofectamine™ 3000 (Thermo Fisher Scientific, Waltham, MA, United States). The siRNAs and inhibitor sequences were as follows: si-*Linc00239* sense: 5′-GCUGGCAAAUAUGAAGGUATT-3′, antisense: 5′-GCUGGCAAAUAUGAAGGUATT-3′; si-*Linc00239* NC sense: 5′-UUCUCCGAACGUGUCACGUTT-3′, antisense: 5′-ACGUGACACGUUCGGAGAATT-3′; miR-204-5p inhibitor: 5′-GGCUACAGUCUUUCUUCAUGUGACUCGUGGACUUCCCUUU-3′; miR-204-5p inhibitor NC: 5′-CAGUACUUUUGUGUAGUACAA-3′.

### Cell Counting Kit-8

Cell proliferation was determined using Cell Counting Kit-8 (CCK-8). Briefly, the cell suspensions of 786-O and SN12C at a density of 2 × 10^4^ were seeded following transfection for 48 h in a 96 well plate, after which the CCK-8 regent was added. At 24, 48, and 72 h, Elx800 (BioTek, Winooski, Vermont, USA) was used to observe absorbance (450 nM).

### Transwell assay

Before conducting the invasive experiment, Matrigel (Biosciences, Sparks, MD) was placed into a 24 well Transwell plate (the upper chamber) to precoat the membrane. Cell suspensions of 786-O and SN12C at a concentration of 2 × 10^5^ were seeded in the upper chamber (100 μl/well). Additionally, 600 μl DMEM (Thermo Fisher Scientific) or RPMI1640, including 10% FBS, were delivered to the lower compartment of each Transwell plate. Cells were fixed with methanol (Sangon biotech, Shanghai, China) at 25 °C for 30 min following 48 h of incubation. Subsequently, 0.1% crystal violet (Sangon biotech) was utilized to visualize cells for 20 min. The migrated and invaded cells were visualized using an inverted microscope (Optec, Chongqing, China) after the non-migrated cells were wiped off.

### Quantitative real-time polymerase chain reaction

Total RNA extraction was carried out using Trizol (Thermo Fisher Scientific). Following the instructions of the PrimeScript™ RT reagent kit (Takara, DaLian, China), cDNA was obtained. Quantitative real-time polymerase chain reaction (qRT-PCR) was performed on ABI 7500 using TB Green Fast qPCR Mix (Takara). The conditions were as follows: 95 °C for 30 s; 40 cycles of 95 °C for 30 s and 60 °C for 20 s. The primers are shown in Table 1. When calculating lncRNA and mRNA expression, GAPDH was used as a control. When calculating the relative expression of microRNA, U6 was used as a control.

**Table 1:** The primers in this study

Primer name	Sequence (5′-3′)
linc00239-F	AGGTACCAGCTGGGATGTTG
linc00239-R	CATGACCGTCTTCCTGTGTG
RAB22A-F	GCACCAATGTACTATCGAGGGTC
RAB22A-R	CATGCTGTCGAAGCTCTTTCACC
GAPDH-F	GAGTCAACGGATTTGGTCGT
GAPDH-R	GACAAGCTTCCCGTTCTCAG
miR-204-5p-F	AACACGCTTCCCTTTGTCATC
miR-204-5p-RT	GTCGTATCCAGTGCAGGGTCCGAGGTATTCGCACTGGATACGACAGGCAT
miR-26b-5p-F	AGCCAGCGTTCAAGTAATTCA
miR-26b-5p-RT	GTCGTATCCAGTGCAGGGTCCGAGGTATTCGCACTGGATACGACACCTAT
miR-203a-3p-F	AAGCGACCGTGAAATGTTTAGG
miR-203a-3p-RT	GTCGTATCCAGTGCAGGGTCCGAGGTATTCGCACTGGATACGACCTAGTG
miR-320a-F	AACACGTGAAAAGCTGGGTTG
miR-320a-RT	GTCGTATCCAGTGCAGGGTCCGAGGTATTCGCACTGGATACGACTCGCCC
UNIVERSE-R	GTGCAGGGTCCGAGGT
U6-F	CTCGCTTCGGCAGCACA
U6-R	AACGCTTCACGAATTTGCGT

### Western blot analysis

First, siRNAs or the miR-204-5p inhibitor were transfected for the indicated time after washing with phosphate-buffered saline (Sigma-Aldrich, St. Louis, United States) twice in SN12C and 786-O cells. 1 × 10^6^ cells were then treated with RIPA lysis buffer (Beyotime, Shanghai, China) containing 1 mM PMSF (Sigma-Aldrich), followed by centrifugation at 10,000 × *g* for 5 min. Next, the proteins (30 μg/sample) in the supernatant were further separated via electrophoresis with sodium dodecyl sulfate–polyacrylamide gel electrophoresis. Then, the proteins were transferred from the gel to a polyvinylidene fluoride membrane (Sangon biotech). Subsequently, the membranes were incubated with rabbit monoclonal antibody against *RAB22A* (Abcam, Cambridge, Cambs, Britain, #ab137093) and rabbit polyclonal antibody against GAPDH (Proteintech, Wuhan, China, #10494-1AP) overnight, followed by incubation with horseradish peroxidase-conjugated goat anti-rabbit secondary antibody (Cusabio, Wuhan, China, #CSB-PA489724) for 2 h. The membranes were finally developed and the protein bands were visualized using ECL reagents (ThermoFisher Scientific) and Tanon-5200CE (Biotanon, China).

### Dual-luciferase reporter gene assay

Dual-luciferase reporter plasmids were created using pmirGLO dual-luciferase vectors from Promega (Madison, WI, USA). The relevant plasmid (200 ng/well) and miRNA (400 ng/well) were co-transfected into 1 × 10^5^ HEK-293 T cells in a 24 well plate for 48 h. The dual-luciferase reporter kit from Promega was used to measure luciferase activity. Renilla luciferase activity was used to standardize the relative activity of firefly luciferase.

### Statistical analysis

Statistical analysis was conducted using Graphpad 8.0 and SPSS 10.0 software (SPSS Inc., IL, USA). Significance levels were assessed using *t-*test and one-way analysis of variance, followed by the Dunnett test. All data were presented as mean ± standard deviation. All experiments were performed in triplicate.

## Results

### Elevated Linc00239 in ccRCC leads to poor prognosis

**Fig. 1:**
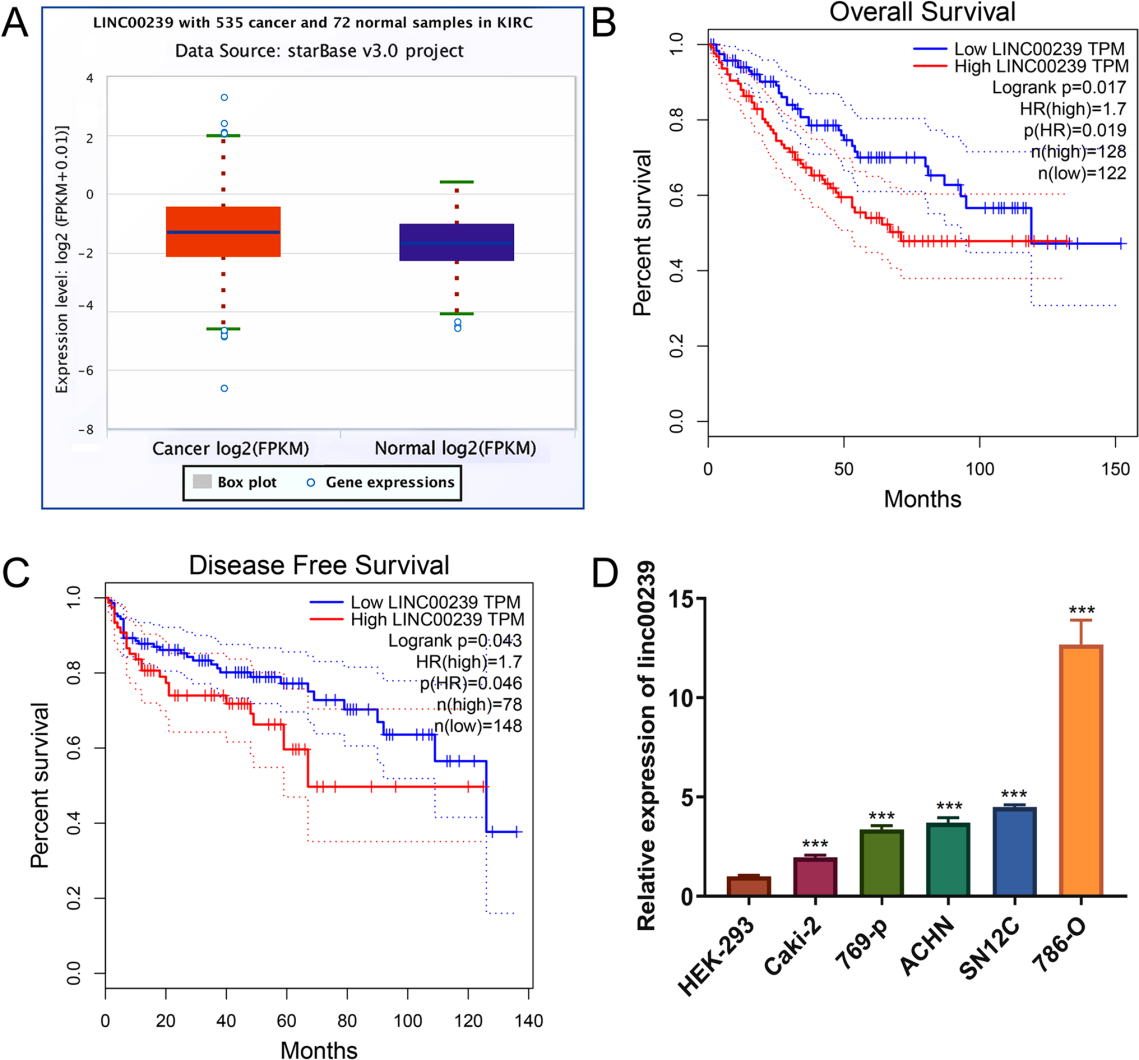
Elevated *Linc00239* in ccRCC is correlated with poor prognosis. **A** *Linc00239* expression levels are elevated in ccRCC tissues, as analyzed using StarBase. **B** Overall survival is decreased under high *Linc00239* expression levels, as per data obtained from TCGA. **C** Disease-free survival is decreased under high *Linc00239* expression levels*,* as per data from TCGA. **D** *Linc00239* is highly expressed in ccRCC cells (Caki-2, ACHN, 769-p, SN12C, and 786-O) with HEK-293 as a control. The expression levels of *Linc00239* in ccRCC and HEK-293 cells were evaluated using qRT-PCR; n = 3, ***P < 0.001

We first analyzed the data obtained from StarBase, which quantified *Linc00239* levels in 535 cancer and 72 normal tissues. *Linc00239* was overexpressed in ccRCC (Fig. 1A). We also conducted a prognosis analysis between high and low *Linc00239* levels in ccRCC using TCGA. The overall and disease-free survivals were better reflected in the group with high *Linc00239* levels than in the group with low *Linc00239* levels (Fig. 1B, C). Additionally, we determined *Linc00239* expression in ccRCC lines via qRT-PCR using HEK-293 as a control. As shown in Fig. 1D, *Linc00239* was overexpressed in ccRCC lines than in HEK-293 cells, consistent with the observations obtained from the tissues. Among them, 786-O cells exhibited the highest expression of *Linc00239* followed by SN12C cells, while Caki-2 cells exhibited the lowest expression of *Linc00239* followed by 769-p cells (Fig. 1D). Conclusively, our results indicate that *Linc00239* demonstrates high expression alongside poor prognosis in ccRCC.

### Proliferation, migration, and invasion were repressed in Linc00239-knockdown ccRCC cells

**Fig. 2:**
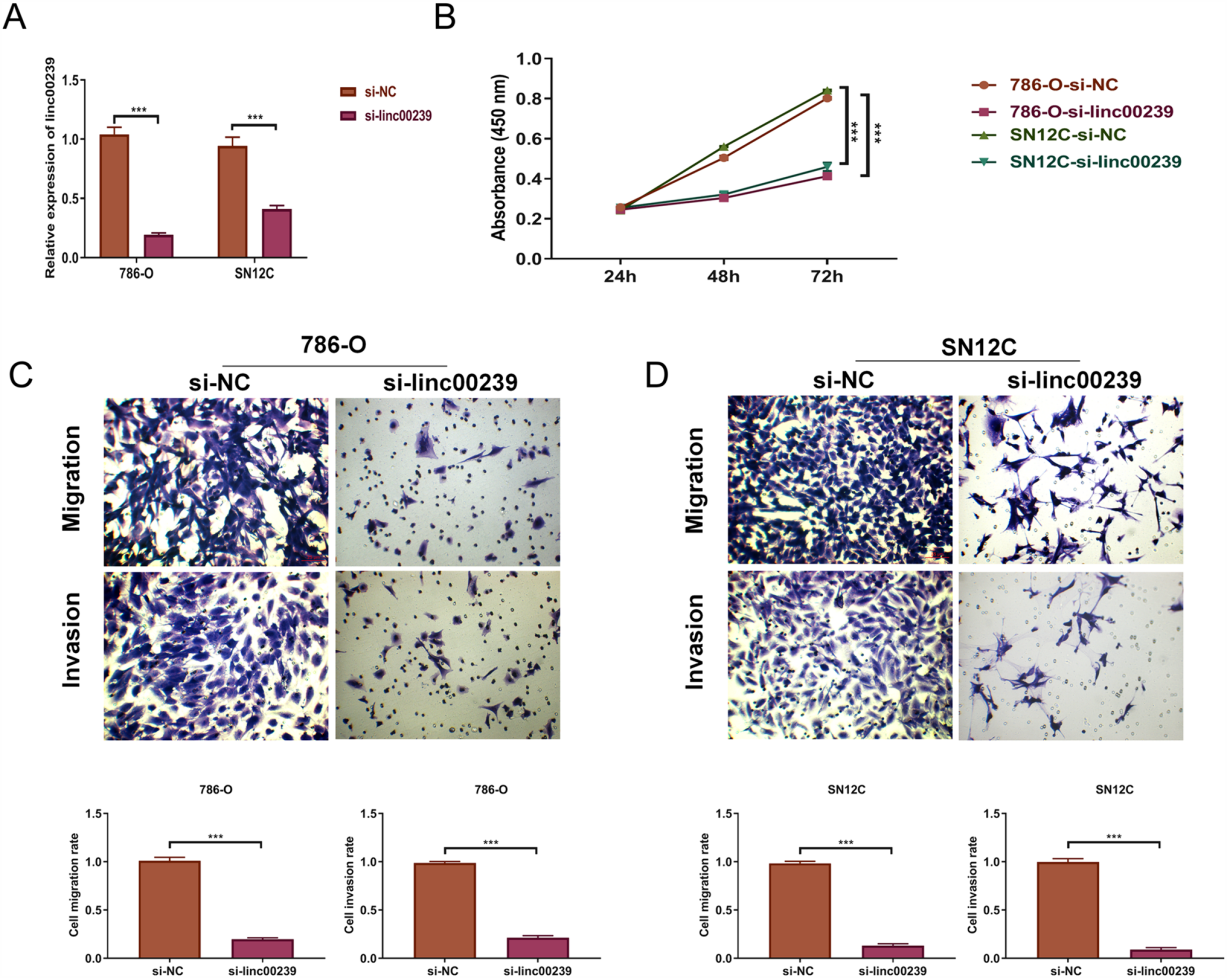
Repressed proliferation, migration, and invasion in *Linc00239*-knockdown cells. **A** *Linc00239* knockdown was effectively performed in SN12C and 786-O cells. *Linc00239* expression levels were determined using qRT-PCR after transfecting with si-NC and si-*Linc00239* for 48 h. **B** *Linc00239* inhibition decreased cell proliferation in 786-O and SN12C cells. CCK-8 was used to determine cell proliferation. In 786-O (**C**) and SN12C (**D**) cells, *Linc00239* inhibition suppressed cell migration and invasion. The migration and invasion abilities of the cells were measured using Transwell assay. si-NC: siRNAs of *Linc00293* negative control; si-linc00293: siRNAs of *Linc00293*; n = 3, ***P < 0.001

We selected SN12C and 786-O cells for subsequent analyses as they exhibited the highest expression levels of *Linc00239*. First, *Linc00239* expression was silenced in the cells by transfecting siRNAs with *Linc00239* (si-*Linc00239* group) and its negative control (si-NC group) for 48 h. As shown in Fig. 2A, *Linc00239* levels markedly decreased following transfection into siRNAs for 48 h. *Linc00239* levels decreased by > 50% in the si-*Linc00239* group compared with the si-NC group in SN12C and 786-O cells (Fig. 2A). On further comparing cell proliferation, we found that *Linc00239* silencing markedly repressed cell proliferation in both SN12C and 786-O cells at 48 and 72 h (Fig. 2B). Subsequently, a Transwell assay was used to measure the effect of *Linc00293* silencing on migration and invasion (Fig. 2C, D), which showed that both migration and invasion were inhibited by *Linc00293* knockdown in SN12C and 786-O cells. Collectively, silencing *Linc00239* repressed cell proliferation, migration, and invasion.

### Linc00239 regulates miR-204-5p/RAB22A *axis* in ccRCC cells

**Fig. 3:**
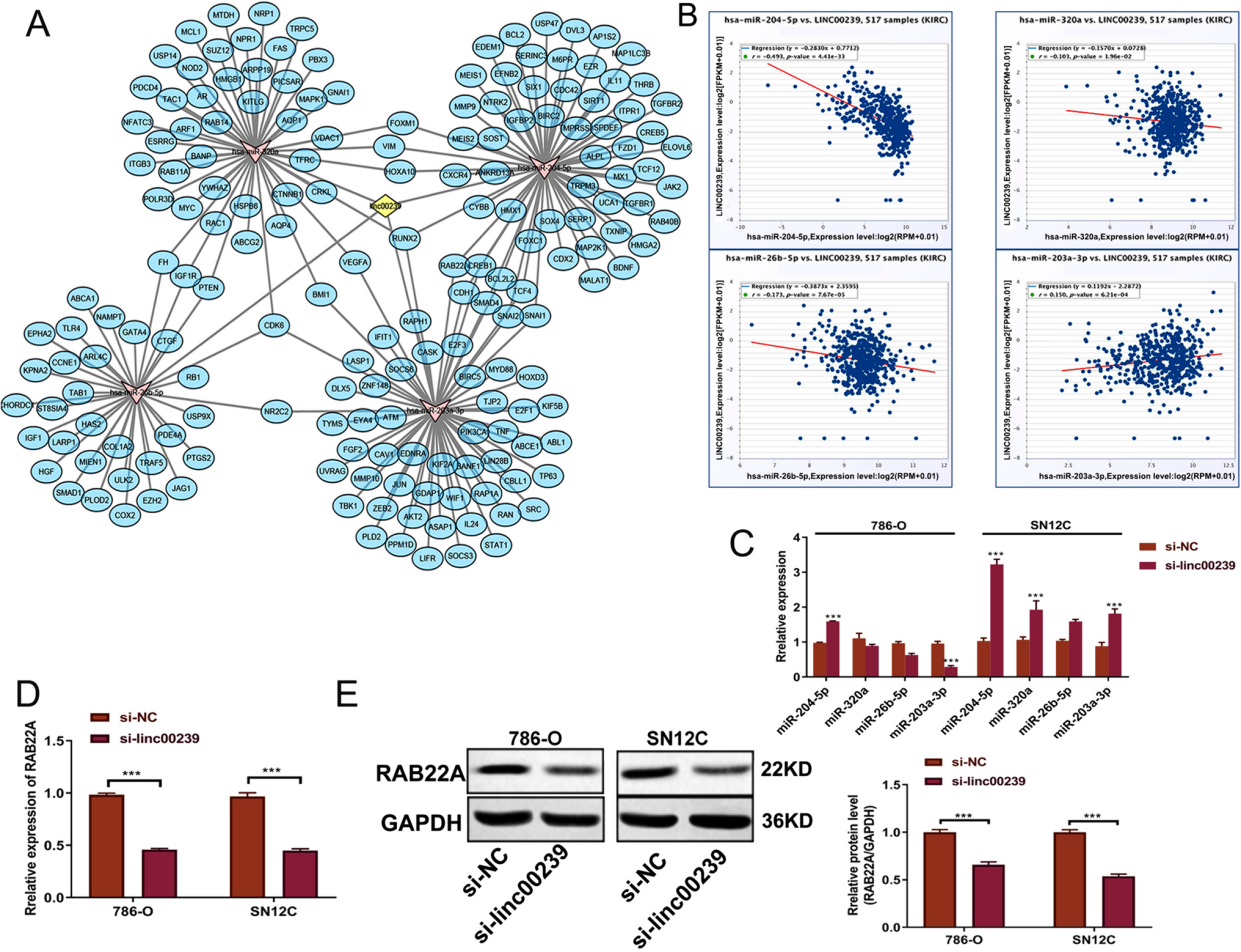
*Linc00239* regulates the miR-204-5p/*RAB22A* axis in cells. **A** *Linc00239*–microRNA–mRNA network was constructed using Cytoscape based on the prediction of the miRanda and miTarbase databases. Yellow indicates *Linc00239*, pink indicates the four selected microRNAs, and blue indicates mRNA potential regulated by the microRNAs. **B** Relationship between *Linc00239* and miR-320a, miR-26b-5p, miR-203a-3p, and miR-204-5p was drawn using StarBase. Among the four microRNAs, the levels of miR-204-5p, miR-26b-5p, miR-320a were repressed, while that of miR-203a-3p was elevated when *Linc00239* was overexpressed. **C** miR-320a, miR-26b-5p, miR-203a-3p, and miR-204-5p levels in 786-O and SN12C cells after silencing *Linc00239* were measured via qRT-PCR. Only miR-204-5p exhibited the same tendency in both the cell lines and is upregulated after *Linc00239* knockdown. **D**, **E** miR-204-5p targeted *RAB22A*, which was decreased after silencing *Linc00239*. *RAB22A* was assessed using qRT-PCR (**D**) and western blot analysis (**E**). *RAB22A*: Ras-related proteins in brain 22a; n = 3, ***P < 0.001

To uncover the mechanism of *Linc00239* in ccRCC, we first analyzed the potential microRNA regulated by *Linc00239* based on the predictions of the miRanda and MiRTarBase databases. Four microRNAs involved in ccRCC were selected based on previous studies [17–20], namely, miR-204-5p, miR-26b-5p, miR-203a-3p, and miR-320a. Of these microRNA, the levels of miR-204-5p, miR-26b-5p, and miR-320a decreased, while that of miR-203a-3p increased in ccRCC [17–20]. Finally, the *Linc00239*–microRNA–mRNA network was constructed using Cytoscape. As shown in Fig. 3A, yellow indicates *Linc00239*, pink indicates the four selected microRNAs, and blue indicates the mRNAs potentially regulated by the microRNAs. In addition, we analyzed the connection between *Linc00239* and the four microRNAs using StarBase. Among the four microRNAs, the expression levels of miR-204-5p, miR-26b-5p, and miR-320a were repressed and that of miR-203a-3p was elevated when *Linc00239* was overexpressed (Fig. 3B). To verify whether *Linc00239* regulates the expression levels of the four microRNAs, the expression levels of the microRNAs following *Linc00239* inhibition were determined using qRT-PCR. We found that miR-204-5p was elevated while miR-203a-3p was markedly suppressed in *Linc00239*-knockdown 786-O cells. However, the levels of miR-320a, miR-26b-5p, miR-203a-3p, and miR-204-5p were elevated following *Linc00239* inhibition in SN12C cells. Of these four microRNAs, only miR-204-5p exhibited the same tendency in 786-O and SN12C cells after silencing *Linc00239* (Fig. 3C). Therefore, *Linc00239* regulates the level of miR-204-5p in ccRCC (Fig. 3B, C). As reported by previous studies, miR-204-5p is repressed in ccRCC, and its level correlates negatively with that of *RAB22A*. Meanwhile, miR-204-5p regulates *RAB22A* expression by directly binding with *RAB22A* [19]. Accordingly, we further monitored the expression levels of *RAB22A* in *Linc00239*-knockdown SN12C and 786-O cells. Our results showed that *RAB22A* exhibited greater suppression at the gene and protein levels in the si-*Linc00239* group than in the si-NC group (Fig. 3D, E). These results demonstrate that in ccRCC, *Linc00239* regulates the miR-204-5p/*RAB22A* axis.

### Linc00239 regulates the proliferation, migration, and invasion of ccRCC cells via the miR-204-5p/RAB22A *axis*

**Fig. 4:**
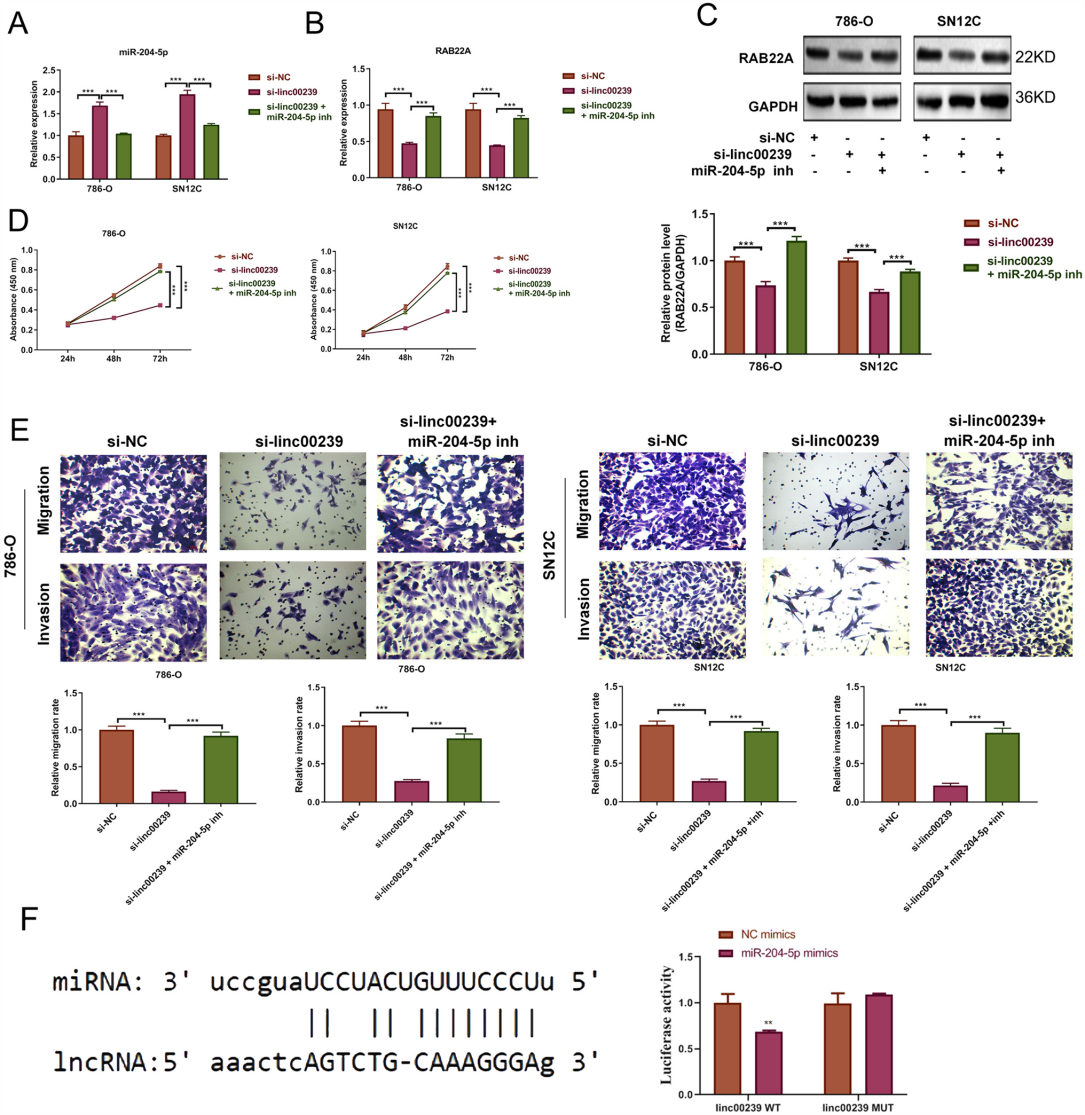
*Linc00239* regulates proliferation, migration, and invasion via the miR-204-5p/*RAB22A* axis. **A** qRT-PCR of miR-204-5p level. **B** *RAB22A* mRNA was analyzed using qRT-PCR. **C** *RAB22A* protein was assessed via western blot analysis. **D** CCK-8 analysis of cell proliferation. **E** Migration and invasion abilities were evaluated using Transwell assay. **F** The predicted binding site between miR-204-5p and LINC00239 (left); Dual luciferase assay confirmed that miR-204-5p binds to the predicted binding site of LINC00239 (right). *RAB22A*: Ras-related proteins in brain 22a; n = 3, ***P < 0.001

To confirm that *Linc00239* regulates cell function through the miR-204-5p/*RAB22A* axis, we synthesized the miR-204-5p inhibitor and cotransfected them with si-*Linc00239* (si-*Linc00239* + miR-204-5p inhibitor). miR-204-5p levels were elevated and those of *RAB22A* were suppressed in the *Linc00239*-knockdown SN12C and 786-O cells ( Fig. 3) compared with the negative control cells (Fig. 4A–C). However, silencing miR-204-5p reduced the miR-204-5p levels, indicating the effectiveness of the miR-204-5p inhibitor. Moreover, the expression of the target gene *RAB22A* was upregulated after silencing miR-204-5p. We also evaluated cell proliferation. As shown in Fig. 4D, silencing miR-204-5p rescued the inhibiting effect on cell proliferation when *Linc00239* was knocked down. Moreover, silencing miR-204-5p attenuated the inhibitory effect of *Linc00239*-knockdown cells on migration and invasion (Fig. 4E). In addition, miR-204-5p mimics significantly inhibited the luciferase activity of *Linc00239*-WT, but the inhibition of the luciferase activity of *Linc00239*-WT was eliminated upon mutation of the probable miR-204-5p binding site (Fig. 4F). These results confirm that *Linc00239* regulates cell proliferation, migration, and invasion via the miR-204-5p/*RAB22A* axis.

## Discussion

*Linc00239* is overexpressed in CRC, which promotes cell progress via direct binding to microRNA-484 and regulates KLF12 expression [14]. *Linc00239* is also overexpressed in acute myeloid leukemia cells and promotes oncogenic behavior by regulating PI3K/AKT/mTOR signaling [16]. Consistent with previous findings, this study showed that *Linc00239* is highly expressed in ccRCC. However, Ye et al. suggested that *Linc00239* is a predictor of hepatocellular carcinoma, and its high expression indicates a better recurrence-free and overall survival through risk scoring [21, 22]. Our results demonstrated that *Linc00239* exhibits a high expression with a poor prognosis in ccRCC, contrary to those of a previous study, attributed to the tissue specificity of *Linc00239* expression [23].

Accumulating evidence has shown that miR-26b-5p exerts different roles in different diseases, facilitating lung cancer and gallbladder carcinoma progression and suppressing human papillary thyroid cancer, Burkitt lymphoma, and ccRCC development [17, 24–27]. Herein, miR-26b-5p expression levels were suppressed in 786-O cells but elevated in SN12C cells after silencing *Linc00239*. miR-320a was also reported to play different roles in various cancers, promoting the progress in CRC while inhibiting the progress in nasopharyngeal carcinoma cells, lung cancer, and ccRCC [28–31]. In this study, miR-320a expression levels were suppressed in 786-O cells and elevated in SN12C cells when *Linc00239* was knocked down. miR-204-5p demonstrates conflicting effects in different cancers, exerting a promoting role in cholangiocarcinoma and an inhibiting role in gastric cancer and ccRCC [19, 32, 33]. Herein, miR-204-5p expression levels were promoted in ccRCC when *Linc00239* was knocked down. miR-203a-3p has been reported as a promotion factor in ccRCC, whereas it is an inhibition factor in breast carcinoma and pancreatic cancer [20, 34, 35]. In this study, miR-203a-3p expression levels were significantly decreased in 786-O cells but increased in SN12C cells when *Linc00239* was knocked down. Therefore, only miR-204-5p exhibits the same expression trends in both the cell lines. *Linc00239* knockdown inhibits cell progression but increases the expression levels of miR-204-5p. These results indicate that *Linc00239* knockdown inhibits ccRCC progress by upregulating the expression levels of miR-204-5p, consistent with the findings of a previous study [19]. Therefore, miR-204-5p was selected for further analyses.

*RAB22A* belongs to the Ras superfamily, which reportedly has > 60 members [36]. *RAB22A* was reported as an oncogenic gene in breast cancer, CRC, osteosarcoma, and ccRCC [19, 36–38]. Previous studies have reported that miR-204-5p targets the *RAB22A* gene [19]. Our results showed RAB22A levels were positively correlated with *Linc00239* and negatively correlated with miR-204-5p, suggesting that *RAB22A* is an oncogene in ccRCC.

Taken together, in ccRCC tissues and cells, elevated *Linc00239* denotes a poor prognosis. Inhibiting *Linc00239* levels markedly reduced cell proliferation, migration, and invasion. However, the miR-204-5p inhibitor reversed *Linc00239* knockdown and suppressed the decrease in proliferation, migration, and invasion. *RAB22A* expression levels were decreased when *Linc00239* was silenced but significantly increased when transfecting miR-204-5p inhibitor to *Linc00239*-knockdown cells. These results indicate that *Linc00239* may accelerate ccRCC progression through the miR-204-5p/RAB22A axis, which provides novel clues for diagnosing and treating ccRCC. However, *Linc00239*, *RAB22A*, and miR-204-5p expression levels need further analysis to illuminate the relationship between them and their clinical features in more ccRCC tissues. The mechanism of *Linc00239* in ccRCC requires further verification with in vivo and in vitro experiments.

## Data Availability

Datasets generated and/or analyzed during research are available in [StarBase] repository (http://StarBase.sysu.edu.cn/panGeneDiffExp.php) and [TCGA] repository (http://gepia.cancer-pku.cn/detail.php?gene=Linc00239###).
